# AI-Powered Simulation for Nursing Education: Mixed Methods Systematic Review

**DOI:** 10.2196/95167

**Published:** 2026-07-21

**Authors:** Hongzhan Jiang, Ziyan Wang, Wanting Shen, Meiqi Meng, Dan Yang, Xuejing Li, Yufang Hao

**Affiliations:** 1 School of Nursing, Beijing University of Chinese Medicine, Liangxiang University Town, Fangshan District, Beijing, 100000, China, 86 18911091028

**Keywords:** artificial intelligence, nursing education, simulation training, virtual reality, mixed methods, systematic review

## Abstract

**Background:**

Traditional simulation-based nursing education is often constrained by high costs, resource intensity, and limited scalability. AI-powered simulations offer dynamic, scalable, and personalized alternatives. However, the empirical evidence regarding their pedagogical effectiveness and learner acceptance remains fragmented.

**Objective:**

This study aimed to systematically evaluate and synthesize evidence on the effectiveness and learner perceptions of AI-powered simulations in nursing education.

**Methods:**

Following PRISMA (Preferred Reporting Items for Systematic Reviews and Meta-Analyses) guidelines, we systematically searched 11 electronic databases (PubMed, CINAHL, Embase, Web of Science, Cochrane Library, Scopus, SinoMed, CNKI, Wanfang, VIP, and Google Scholar) for studies published between January 2014 and September 2025. Two independent reviewers performed study selection, data extraction, and quality appraisal using design-specific tools (risk of bias 2 tool [RoB 2; Cochrane Bias Methods Group] for randomized controlled trials [RCTs], Risk Of Bias in Nonrandomized Studies of Interventions [ROBINS-I; Cochrane Bias Methods Group] for nonrandomized studies, Mixed Methods Appraisal Tool [MMAT] for mixed methods, Joanna Briggs Institute [JBI] for qualitative, and Agency for Healthcare Research and Quality [AHRQ] for cross-sectional studies). Quantitative data were synthesized narratively, and qualitative findings were integrated using JBI meta-aggregation. A convergent segregated approach with joint display was used to generate meta-inferences.

**Results:**

Nineteen studies involving 1253 participants (primarily prelicensure nursing students, with some interdisciplinary cohorts) were included. AI modalities comprised generative AI/large language models (n=7), AI-driven virtual patients/mannequins (n=5), AI-enhanced virtual/mixed reality (n=5), and chatbots (n=2). Three studies were RCTs, 4 were quasiexperimental with control groups, 3 were uncontrolled pre-post studies, 4 were mixed methods, 4 were qualitative, and one was a cross-sectional survey. Quantitative synthesis showed that evidence from RCTs and controlled quasiexperimental studies indicates significant improvements in cognitive knowledge and affective outcomes, including self-efficacy and communication confidence; however, effects on complex psychomotor skills were inconsistent, with one RCT finding AI-assisted simulation inferior to standardized patient simulation. Findings from uncontrolled designs are preliminary. Qualitative meta-aggregation revealed that learners valued safe, repeatable, nonjudgmental practice environments that reduced anxiety and bridged the theory-practice gap. Persistent challenges included technical frustrations, “robotic” interactions, lack of nonverbal cues, and system instability, collectively constituting an “authenticity gap.”

**Conclusions:**

AI-powered simulations show promise for developing foundational clinical reasoning and communication skills in nursing education, though the evidence base is limited by the predominance of uncontrolled designs, reliance on self-reported measures, and absence of longitudinal data on skill retention or clinical transfer. Due to current technological limitations in replicating physical and emotional authenticity, AI should be implemented as a complementary tool alongside traditional simulation methods and clinical placements, rather than as a replacement. Future research should prioritize longitudinal outcomes, standardized competency measures, RCTs with active comparators, and implementation strategies addressing technical barriers.

## Introduction

Nursing education faces unprecedented challenges in the 21st century, with increasing patient complexity, rapid technological advancement, and the critical need to bridge the theory-practice gap while ensuring patient safety [1,2]. Traditional clinical training models, which rely heavily on direct patient contact, are constrained by limited clinical placement opportunities, ethical considerations, variability in clinical experiences, and concerns about patient safety during the learning process [3,4]. Consequently, simulation-based education has emerged as an essential pedagogical strategy to provide standardized, repeatable, and safe learning environments for nursing students [5].

Simulation-based learning in nursing has evolved significantly over the past 2 decades, progressing from simple task trainers to sophisticated high-fidelity manikin-based simulations [6]. These approaches have demonstrated effectiveness in improving clinical competence, critical thinking, and clinical decision-making skills [7,8]. However, conventional simulation methods face inherent limitations, including high costs, resource intensity, the need for trained facilitators, scheduling constraints, and limited scalability [9,10]. Moreover, traditional simulations often follow predetermined scripts with limited adaptability to individual learner needs and learning trajectories [11].

The rapid advancement of AI technologies has opened new frontiers in health care education [12]. AI-powered simulations leverage machine learning algorithms, natural language processing, virtual reality (VR), and adaptive learning systems to create dynamic, personalized, and intelligent learning environments [13,14]. These technologies enable real-time feedback, adaptive scenario complexity, automated performance assessment, and data-driven learning analytics that were previously unattainable [15,16]. Early applications have demonstrated promise in medical education, including surgical training, diagnostic reasoning, and patient communication skills development [17,18].

Recent years have witnessed a growing integration of AI technologies into nursing education simulations, including AI-powered virtual patients, intelligent tutoring systems, conversational agents, augmented reality applications, and predictive learning analytics platforms [19,20]. These innovations potentially address the limitations of traditional simulation by offering cost-effective scalability, 24/7 accessibility, personalized learning pathways, objective assessment capabilities, and immediate adaptive feedback [21,22]. Preliminary studies suggest that AI-enhanced simulations may improve knowledge retention, clinical reasoning, technical skills, and learner engagement [23,24].

Despite the proliferation of AI-powered simulation technologies in nursing education, the evidence base remains fragmented and characterized by considerable heterogeneity in AI modalities, study designs, and outcome measures [25,26]. For the purposes of this review, we classify AI-powered simulations into four categories distinguished by their technical architectures and pedagogical mechanisms: (1) generative AI (GenAI)/large language models (LLMs); (2) AI-driven virtual patients/mannequins; (3) AI-enhanced VR/mixed reality (MR); and (4) AI chatbots/tutors. While these modalities differ in technological implementation, they share a common pedagogical mechanism, including the use of AI to drive interactive, adaptive, and real-time learning experiences that distinguish them from static or rule-based simulations [27].

Critical questions remain unanswered: “What types of learning outcomes are most effectively enhanced by AI-powered simulations?” “How do these technologies compare to traditional simulation methods?” “Which specific AI features contribute most to learning effectiveness?” [28,29]. Furthermore, while several narrative reviews have discussed the potential of technology-enhanced learning in nursing [30,31], no comprehensive systematic review has specifically synthesized the empirical evidence regarding the impact of AI-powered simulations on nursing education learning outcomes, with explicit attention to evidence quality and risk of bias. Such a synthesis is urgently needed to inform evidence-based educational practice, guide future technology development, and identify research priorities in this rapidly evolving field.

This mixed methods systematic review aims to comprehensively evaluate and synthesize the current evidence on AI-powered simulation interventions in nursing education and their impact on learning outcomes. Specifically, this review seeks to (1) identify and categorize the types of AI technologies used in nursing simulation education; (2) systematically assess the effects of AI-powered simulations on cognitive, psychomotor, and affective learning outcomes; (3) compare the effectiveness of AI-powered simulations with traditional simulation and conventional teaching methods; and (4) identify gaps in current evidence and provide recommendations for future research and practice. By providing a comprehensive synthesis of the existing evidence, this review will contribute to the growing body of knowledge on technology-enhanced nursing education and inform stakeholders about the potential benefits and limitations of AI-powered simulation in nursing education.

## Methods

### Study Design

This systematic review was conducted in accordance with the PRISMA (Preferred Reporting Items for Systematic Reviews and Meta-Analyses; Checklist 1) guidelines. The review protocol was registered in the PROSPERO (International Prospective Register of Systematic Reviews; registration number: CRD420251208020). A convergent segregated mixed methods systematic review design was used.

### Search Strategy

A comprehensive literature search was performed across multiple electronic databases, including PubMed, CINAHL, Embase, Web of Science, SinoMed, Cochrane Library, CNKI, Wanfang database, Google Scholar, VIP database, and Scopus, from January 1, 2014, to September 30, 2025. The search strategy combined keywords and Medical Subject Headings related to three core concepts: (1) AI, (2) simulation, and (3) nursing education. Sample search terms included “artificial intelligence,” “machine learning,” “AI,” “virtual patient,” “intelligent tutoring system,” “simulation,” “nursing education,” “nursing student,” and “learning outcomes.” The full search strategy for PubMed is provided in Multimedia Appendix 1.

### Inclusion and Exclusion Criteria

Studies were included if they met the following criteria: (1) population: nursing students at any educational level (undergraduate, postgraduate, or continuing education) and practicing nurses enrolled in formal educational activities, consistent with a broad definition of nursing education that encompasses both prelicensure training and lifelong professional development. Studies that enrolled mixed cohorts of health professions learners (eg, medical, nursing, and physician assistant students) were eligible if nursing students constituted a defined subgroup and the educational intervention was situated within a nursing-relevant context; (2) phenomenon of interest: use of AI-powered simulation, defined as any simulation modality in which AI algorithms (machine learning, natural language processing, computer vision, GenAI, adaptive systems, or expert systems) actively drive patient behavior, physiological responses, scenario progression, feedback, or debriefing. This includes conversational virtual patients, AI-enhanced VR, adaptive manikins with AI decision engines, and GenAI clinical scenarios; (3) context: any nursing educational setting (university, hospital-based school, simulation center, or online/blended learning environment); (4) outcomes (quantitative): clinical competence, knowledge, skill performance, critical thinking, clinical judgment, self-efficacy, confidence, anxiety, satisfaction, or transfer to practice measured by validated instruments or objective performance scores; (5) outcomes (qualitative): learner or educator experiences, perceptions of realism, usability, acceptability, facilitators, and barriers; (6) study design: randomized controlled trials (RCTs), quasiexperimental studies, cohort studies, mixed methods studies, and qualitative studies (phenomenology, grounded theory, qualitative description, and thematic analysis); (7) article type: original, empirical, or peer-reviewed journal publications; and (8) language: written in English or Chinese.

Exclusion criteria were (1) studies not involving nursing education; (2) simulation using AI only for debriefing analytics without real-time interaction; (3) studies using only rule-based (non-AI) high-fidelity manikins or static virtual patients; (4) conference abstracts, editorials, and protocols; and (5) studies published before January 1, 2014 (to ensure inclusion only of modern AI technologies post–deep learning revolution).

### Study Selection

All records were imported into NoteExpress (4.2; Beijing Aegean Software Co, Ltd) software. After duplicate removal, titles and abstracts were independently screened by 2 reviewers (HJ and ZW). Full texts were retrieved and assessed against eligibility criteria by the same 2 reviewers (HJ and ZW), with disagreements resolved by a third senior reviewer (XL).

### Data Extraction

Data extraction was conducted independently by 2 reviewers (HJ and ZW) using standardized Joanna Briggs Institute (JBI) templates adapted for this review.

The following data were extracted: authors, year of publication, country, study design, sample size, participant characteristics (level of nursing education, age, and gender), detailed description of the AI-powered simulation intervention using the Template for Intervention Description and Replication (TiDier) checklist [32], comparator condition, outcome measures, key findings, and author conclusions.

All extracted data were cross-checked between the 2 reviewers (HJ and ZW), with discrepancies resolved by consensus or consultation with the third reviewer (XL). Study authors were contacted for missing data or clarification where necessary.

### Risk of Bias (Quality) Assessment

Two reviewers (HJ and WS) independently assessed the methodological quality of all included studies. Disagreements were resolved by consensus or arbitration by a third reviewer (XL).

RCTs were assessed using the Cochrane risk of bias 2 tool (RoB 2). Each domain was judged as low risk, having some concerns, or high risk of bias. An overall risk-of-bias judgment was generated for each study.

Nonrandomized studies (quasiexperimental, pre-post, cohort, and interrupted time-series designs) were evaluated using the Risk of Bias in Nonrandomized Studies of Interventions (ROBINS-I) tool [33]. Bias was assessed across 7 domains (confounding, participant selection, classification of interventions, deviations from intended interventions, missing data, measurement of outcomes, and selection of reported results), with overall judgment categorized as low, moderate, serious, or critical risk of bias.

For cross-sectional studies, the 11-item checklist from the Agency for Healthcare Research and Quality (AHRQ) was used, with items rated as “Yes,” “No,” or “Unclear.” Scores of 0‐3, 4‐7, and ≥8 were classified as representing low, moderate, and high quality, respectively.

For qualitative studies, the JBI Critical Appraisal Checklist (10 items) for qualitative research [34] was used to appraise the study’s philosophical perspective, methodology, design, participant representation, data collection, analysis, and interpretation.

For mixed methods studies, the Mixed Methods Appraisal Tool (MMAT) [35] was used to evaluate the appropriateness of the qualitative, quantitative, and mixed methods components, as well as the overall integration of the findings.

### Data Synthesis

The studies included in the analysis showed excessive heterogeneity, making a formal meta-analysis impossible. We adopted a convergent integrated design, integrating quantitative and qualitative findings through joint display tables and narrative synthesis to derive meta-inferences [36].

## Results

### Search Results

**Figure 1.**
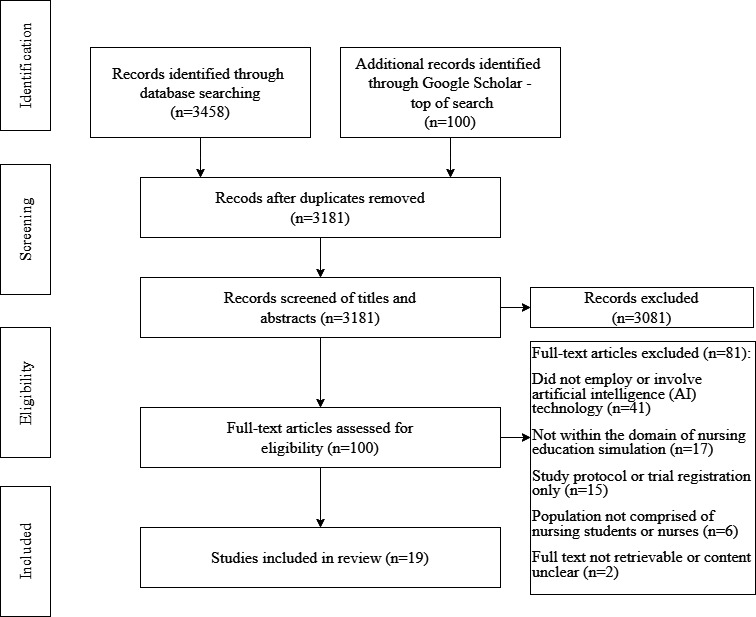
PRISMA (Preferred Reporting Items for Systematic Reviews and Meta-Analyses) flow diagram displaying the process of included records.

Figure 1 illustrates the PRISMA flow diagram of study selection process. After removing duplicates, a total of 3181 citations were identified. Following screening of titles and abstracts, 100 full-text articles were assessed for eligibility based on the inclusion criteria. No additional studies were identified through reference list searching. Ultimately, 19 studies were included.

### Study Characteristics

The 19 included studies comprised a total of 1253 participants. Sample sizes ranged from 10 to 247 participants, and publication years ranged from 2020 to 2025. The 19 studies included 3 (16%) RCTs [37-39], 4 (21%) quasiexperimental studies [40-43], 3 (16%) pre-post studies [44-46], 4 (21%) mixed methods studies [47-50], and 1 (5%) cross-sectional study [51], 4 (21%) qualitative studies [52-55]. Most studies were conducted in the United States (n=5, 26%) [44,46,47,50,51], followed by China (n=4, 21%) [39,40,43,45], Singapore (n=3, 16%) [42,48,53], South Korea (n=2, 11%) [41,49], the United Kingdom (n=2, 11%) [52,55], Turkey (n=1, 5%) [37], Canada (n=1, 5%) [54], and one multinational study (n=1, 5%) [38]. Two studies [40,51] enrolled mixed cohorts. Characteristics of the 19 studies are summarized in Table 1.

**Table 1.** General information of included literature (N=19). Only the participants who completed the AI intervention are included in the total participant count.

Number	Study (Author, Year)	Country	Study design	Sample size (total/grouped)	Participants	Types of AI technologies	Simulation type/platform	Brief description of the intervention	Control group
1	Simsek-Cetinkaya and Cakir (2023) [37]	Turkey	Randomized controlled trial	Total：103 AI-assisted simulation group: 52 Standardized patient simulation group: 51	First-year undergraduate students majoring in nursing	AI-assisted interactive screen-based simulation (AI-AISBS)	Screen simulation+AI avatar	Students conduct simulated breast self-examination (BSE) exercises with AI avatars, and the AI scores the students' performance based on a preset BSE checklist.	Standardized Patient Simulation (SPS) group
2	Fung et al (2025) [38]	Multiple countries (Hong Kong, China; Taiwan, China; Thailand; Spain; South Korea; Australia)	Cross-over randomized controlled trial	Total: 44 Group A (VR^a^→GenAI):^b^ n=22 Group B (GenAI→VR): n=22	Undergraduate nursing students in grades 1-3, from 6 countries/regions, with English as the language of instruction	GenAI	GenAI patient simulation + 360° VR video	GenAI group: students interact with AI patients to collect medical histories, conduct physical examinations, formulate nursing diagnoses and intervention plans, with AI providing real-time feedback and scoring; 360° VR group: watch immersive clinical videos and conduct structured discussions and reflections	360° VR simulation group (cross-over design, all participants experienced both interventions)
3	Chen (2025) [39]	Taiwan, China	Randomized controlled trial	Total: 105 Experimental: n=53 Control: n=52	Undergraduate students majoring in nursing, with an average age of 21.3 years, including 97 females and 8 males.	ChatGPT (4o; OpenAI)	Natural Language Processing VR Communication Simulation (NLP-VRCS)	Students engage in real-time conversations with AI-driven virtual pregnant women through head-mounted displays, covering 3 scenarios: antenatal preparation, childbirth, and postnatal recovery. The system provides immediate feedback and emotional analysis.	Traditional video teaching+role-playing
4	Xiong et al (2025) [40]	China	Quasiexperimental pretest-posttest design	Total: 247 (Questionnaire) Experimental intervention: 38	Clinical nurses and midwives (aged 25-39 years, with work experience ≥3 years)	ChatGPT (OpenAI)	AI-assisted VR Escape Room (360° Panoramic Video + Video Interactive Platform)	VR disaster escape room based on AI-generated scripts, including 4 scenarios: pediatric cardiac arrest, trauma, pneumothorax, and neonatal resuscitation. Participants make interactive decisions via the Bilibili (Bilibili Inc) platform, complete time-limited tasks, and receive feedback.	No independent control group (before-and-after self-control design); in addition, semistructured interviews were conducted with the low-acceptance group (n=25) to explore the influencing factors.
5	Park and Kim (2025) [41]	South Korea	Quasiexperimental study (nonequivalent control group pretest-posttest design)	Total: 72 Experimental: n=38 Control: n=34	A fourth-year student majoring in nursing	AI tutor (Chatbot based on the Danbee [Danbee Inc] platform)	AI tutor-assisted high-fidelity simulation (SimMom)	The experimental group used an AI tutor for puerperal care simulation, including prelearning, simulated practice, and debriefing. The AI provided real-time questions and answers and personalized feedback; 2 hours per week for a total of 5 weeks.	Traditional high-fidelity simulation (without AI tutor)
6	Liaw et al (2025) [42]	Singapore	Wait-list quasiexperimental, type 2 hybrid study trial	Total: 147 Experimental: n=60 Control: n=87	Third-year graduating nursing students from the National University of Singapore, with an average age of 22.6 years old, and 83% being female.	AI doctor (Natural language processing AI based on the Google Cloud Dialogflow engine)	AI-enabled VRS^c^	On the basis of traditional face-to-face simulation, the experimental group was supplemented with AI-enabled VRS. Students conducted interprofessional communication with AI physicians, completed exercises of ABCDE^d^ assessment, clinical decision-making, and communication strategies, and the system provided scoring and feedback.	Traditional face-to-face simulation (without AI-enabled VRS)
7	Chang and Su (2025) [43]	Taiwan, China	Quasiexperimental pretest-posttest design	Total: 66 Experimental: n=33 Control: n=33	Third-year student majoring in nursing	ChatGPT	GenAI-based Patient Character Creation Strategy (GAI-PCC), combined with Xmind (Xmind Ltd) mind mapping software	The experimental group used ChatGPT to assist in creating obstetric patient role scenarios, combined with the self-regulated learning framework (learning motivation, self-management, self-monitoring), to complete evidence-based case analysis and mind map drawing.	Conventional creating personas teaching strategy (C-CPTS), using only Xmind
8	Swan et al (2025) [44]	the United States	Cross-sectional feasibility study with pretest-posttest design	Total: 30 (final sample)	Nursing students (including pre-Bachelor of Science in Nursing [BSN], Master of Nursing [MN], and Doctor of Nursing Practice - Certified Registered Nurse Anesthetist [DNP-CRNA]), with an average age of 28.5 years, 73.3% being female, and 36.7% being African Americans.	AI-enabled mannequin (Human Analog Life-like [HAL] S5301, Gaumard Scientific)	High-fidelity simulation (AI-driven simulated humans)	Students participated in opioid overdose response scenario simulations in groups of 3-4. The AI manikin could respond to voice commands, engage in dialogue, and display physiological responses such as pupil changes and cyanosis. Debriefing was conducted after the simulation.	No control group (feasibility study)
9	Chen and Liou (2025) [45]	Taiwan, China	Single-group pretest and posttest+focus group	Total: 52	Second-year student majoring in nursing	ChatGPT	ChatGPT-driven VR Obstetric Care Communication Simulation System	Students interact with an AI-driven virtual pregnant woman in real time via a head-mounted display, covering 3 scenarios: prenatal, delivery, and postpartum. The system provides real-time voice analysis, emotion recognition, personalized feedback, and scoring.	No control group
10	Anthamatten et al (2025) [46]	United States	Educational project with pretest-posttest design (quasiexperimental, single group)	Total: 85	First-year graduate student in Family Nurse Practitioner (FNP) program	ChatGPT	ChatGPT-driven AI Tutor for oral case presentation (non-VR, screen-based).	Students conducted history-taking with AI virtual patients in groups. Each student then independently gave an oral case presentation to the AI tutor using the Summarize, Narrow, Analyze, Probe, Plan, and Select (SNAPPS) format. The AI system generated quantitative scores and qualitative feedback based on predefined scoring criteria.	No control group
11	Sepanloo et al (2025) [50]	United States	Mixed methods study (platform development+pilot evaluation)	Total: 10 (7 nursing students, 3 nursing teachers)	Nursing students and nursing teachers	Conversational AI	Mixed Reality platform, HoloLens 2 (Microsoft) head-mounted display, digital patients and tools developed through Unity3D (Unity Technologies)	Participants interact with an AI-driven virtual patient via HoloLens 2, simulating 5 time‑phase scenarios of patient physiological deterioration in a hospital setting, where they are required to perform assessment and intervention.	No control group
12	Kim et al (2025) [49]	South Korea	Mixed methods study	Total: 28	Newly recruited nurses (with less than 1 year of experience) and soon-to-graduate nursing students, with an average age of 23.46 years old, 82.1% of whom are female.	GPT-4o	GPT-driven virtual patients enable voice conversations through the VIRTI (Virti Inc) application on head-mounted displays (HMDs)	Participants use an HMD to conduct one-on-one health assessment and communication training with a GPT-powered virtual patient in an acute appendicitis scenario. Each session lasts 1 hour with multiple attempts allowed, followed by a Plus-Delta debriefing to review dialogue transcripts and performance scores.	No control group
13	Liaw et al (2023) [48]	Singapore	Mixed methods study	Total: 32	Graduating students majoring in nursing	AI doctor (Natural language processing AI based on the Google Cloud Dialogflow engine)	Desktop VR Simulation (AI-enabled VRS)	Participants undergo interprofessional communication training with an AI physician via desktop VR across 2 scenarios: sepsis and septic shock.	No control group
14	McGrew et al (2025) [47]	United States	Mixed methods study (pilot implementation+feedback collection)	Total: 10	Midwifery students and dual-degree students of midwifery and family nursing	GenAI	Online AI simulation platform (Re:course AI), Virtual Patient Avatar	Students worked in pairs, alternating between the roles of clinician and observer, and conducted telemedicine simulations via an online platform with t2 AI virtual patients featuring detailed sociocultural backgrounds: Case A – an adolescent from rural United States; Case B – a Somali-American Muslim female.	No control group
15	Carlos Martinez et al (2025) [55]	United Kingdom	Describe qualitative study	Total: 15 (Focus group 1: 7 people, Focus group 2: 8 people)	Second-year student majoring in mental health nursing	AI-driven virtual patients (using 2 interaction methods: menu control and voice control)	The VR platform (Oxford Medical Simulation) is presented through a 2D computer screen.	During the simulated practicum, students interacted with AI-driven virtual patients for history-taking. Each session lasted at least 20 minutes, covering both menu-based and voice-controlled scenarios, with the goal of collecting patient information and making a diagnosis.	No control group
16	Harder et al (2025) [54]	Canada	Qualitative comparative study (focus group)	Total: 240 students participated in the simulation, among whom 20 (4 focus groups) provided qualitative data.	Third-year undergraduate nursing student	AI-enhanced VR (AI-VR	AI-VR (immersive head-mounted display) and standardized patient simulation	Students experienced either AI-VR or standardized patient simulation, with the same scenario (a young female faints, later revealed to have an eating disorder). Both simulations used identical learning objectives and debriefing method (PEARLS)^e^. The AI-VR group interacted with an AI-driven patient via a head-mounted display.	Simulated patient
17	De Mattei et al (2024) [51]	United States	Cross-sectional study design	Total: 135 respondents (22 family nurse practitioner [FNPs], 31 physician assistants [PAs], 43 BSNs, 39 Accelerated Bachelor of Science in Nursing [ABSNs])	Students of FNP, PA, BSN, and ABSN	Artificial Intelligence Virtual Simulated Patient (AI-VSP), with the platform being Patient Communication System (PCS) Spark	Web-based AI virtual patient simulation	During the course, students completed a 10–20 minute AI-VSP simulation (the FNP/PA group completed a “headache” scenario, and the BSN/ABSN group completed an “insomnia” scenario). After the simulation, they completed a questionnaire to evaluate their perceptions of AI-VSP.	No control group
18	Shorey et al (2020) [53]	Singapore	Describe qualitative study	Total: 30 (24 undergraduate nursing students and 6 nursing teachers)	Undergraduate nursing students (participating in virtual patient training) and clinical teachers (assessing students' clinical communication skills)	Artificial Intelligence Virtual Simulated Patient (AI-VSP), with the platform being PCS Spark	AI-based virtual patient simulation (virtual patient)	Prior to clinical placement each semester over a 2-year period (sophomore and junior years), students received communication training using virtual patient simulations across 4 distinct scenarios: a pregnant patient in pain, a patient with recurrent depression, a patient with postoperative wound bleeding, and a peer experiencing internship-related stress. The simulations were conducted in a computer laboratory, with each session lasting approximately 50 minutes. Student experiences were collected via focus groups after the training. Following students’ clinical rotations, clinical instructors were interviewed individually to assess students’ clinical communication skills.	No control group
19	Teixeira et al (2024) [52]	United Kingdom	Qualitative project evaluation (focus group)	Total: 11 (3 focus groups)	Preregistration students in adult nursing	AI-driven virtual patients (including 2 interaction methods: menu-based and voice-controlled)	VR simulation platform	Students experienced 2 types of VR simulations sequentially: menu-based interaction (with Patient Deepak, hypertension) and voice-controlled interaction (with Patient Ray, cluster headache). The system automatically generated a feedback report after each simulation. Students participated in a focus group discussion to share their experiences with the 2 interaction modes.	No control group

a VR: virtual reality.

b GenAI: generative AI.

c VRS: virtual reality simulator.

d ABCDE: airway, breathing, circulation, disability, and exposure

e PEARLS: promoting excellence and reflective learning in simulation.

### Methodological Quality

Three RCTs were included in this systematic review and assessed for quality using the RoB 2 tool. Two of these studies [38,39] were judged to have a low risk of bias, while one [37] was deemed to have some concerns. For the 4 quasiexperimental studies and the 3 pretest-posttest studies, quality assessment was conducted using the ROBINS-I tool. Five of these studies [40,42-44,46] were rated as having a moderate risk of bias, and 2 [41,45] were rated as having a low risk of bias. For the 4 mixed methods studies, the MMAT criteria indicated that 3 studies [48-50] demonstrated high methodological quality, and one study [47] was rated as having moderate methodological quality. For the 4 qualitative studies, the JBI critical appraisal tool was used; 2 [53,54] were assessed as being of moderate quality, and the other 2 [52,55] were of high quality. The one cross-sectional study [51] was evaluated using the AHRQ methodology checklist and was rated as being of moderate quality. Detailed quality assessment results are presented in the Multimedia Appendix 2.

### AI Technologies

Regarding the types of AI technologies used, 7 [38,39,43,45-47,49] studies applied GenAI or LLMs (eg, ChatGPT [OpenAI]), 5 [37,44,52,53,55] studies used AI-driven virtual patients or mannequins, 5 [40,48,50,51,54] studies focused on AI-enhanced VR or MR simulations, and 2 [41,42] studies incorporated AI chatbots as adjunctive tools. In terms of simulation platforms, 7 [37,46,47,51-53,55] out of 19 (37%) studies used screen-based or web-based simulations, 8 [38-40,45,48-50,54] out of 19 (42%) studies used VR or MR with head-mounted displays, 3 [41,42,44] out of 19 (16%) studies used high-fidelity mannequins, and 1 [43] out of 19 (5%) studies integrated AI as a supplement to traditional simulation methods. Concerning control group designs, 8 [37-39,41-44,54] out of 19 (42%) studies included a parallel control group, comparing AI interventions with traditional teaching, standard simulation, or alternative technologies, while 11 [40,45-53,55] out of 19 (58%) studies had no control group, using pretest-posttest designs or qualitative feasibility assessments.

### Outcome

#### Overview

Various measurement methods were used to evaluate the teaching effectiveness of AI simulations (Multimedia Appendix 3). The most frequently used scales included the System Usability Scale (SUS), which was used in 3 studies [40,44,45], the Technology Acceptance Model (TAM) Questionnaire, used in 2 studies [48,49], and the National Aeronautics and Space Administration Task Load Index (NASA-TLX), also used in 2 studies [40,50]. All assessment tools are summarized in Textbox 1.

Textbox 1. **Summary of assessment tools.**

Knowledge/Skills Assessment Tools

Breast self-examination (BSE) checklist

Communication Knowledge Quiz

Maternal and Newborn Care Communication Assessment Form (MNCCAF)

Opioid Overdose Knowledge Scale (OOKS)

Attitude / Self-Efficacy Assessment Tools

Student Satisfaction and Self-Confidence in Learning Scale

Spielberger State-Trait Anxiety Inventory (STAI)

Jefferson Scale of Empathy-Healthcare Providers (JSE-HP)

Communication Confidence Self-Assessment Form (CCSF)

Opioid Overdose Attitudes Scale (OOAS)

Self-Efficacy Scale

Patient Clinical Information Exchange and Interprofessional Communication Self-Efficacy Scale (PIEie-SES)

Technology Acceptance and Usability Assessment Tools

System Usability Scale (SUS)

Technology Acceptance Model (TAM) Questionnaire

Agent Persona Instrument (API)

Chatbot Usability Scale

National Aeronautics and Space Administration Task Load Index (NASA-TLX)

Implementation Outcome Assessment Tools

Acceptability of Intervention Measure (AIM)

Intervention Appropriateness Measure (IAM)

Feasibility of Intervention Measure (FIM)

Clinical Competency/Performance Assessment Tools

Clinical Competence Questionnaire (CCQ)

Gap-Kalamazoo Communication Skills Assessment Form (GKCSAF)

Nursing Performance Profile 5 Instrument

Oral Case Presentation Ability Score

AI Chatbot Assessment Rubric

Situational Awareness Assessment Tool

Situation Awareness Global Assessment Technique (SAGAT)

Qualitative Assessment Tools

Focus Group Interview Guide

Think-Aloud Protocol

Debriefing Questions

#### Knowledge and Skills Assessment

Among the 19 included studies, 11 assessed knowledge or skills outcomes using various validated tools (Textbox 1). Of these, 8 [38,39,43-46,48,50] out of 11 (73%) studies reported improvements following AI-enhanced simulations. These gains were primarily observed in uncontrolled pre-post and quasiexperimental designs. Knowledge gains were demonstrated using the Clinical Competence Questionnaire (CCQ) [38], Obstetric Nursing Knowledge Tests [41,43], Opioid Overdose Knowledge Scale (OOKS) [44], Communication Knowledge Quizzes [42,48], and Post-Case Quizzes [46]. Skills improvements were measured using the breast self-examination (BSE) checklist [37], Maternal and Newborn Care Communication Assessment Form (MNCCAF) [45], Gap-Kalamazoo Communication Skills Assessment Form (GKCSAF) [39], Nursing Performance Profile 5 [50], and Oral Case Presentation Ability Scores [46]. One RCT found that AI-assisted simulation was inferior to standardized patient simulation for BSE skills [37], and 2 studies reported nonsignificant knowledge improvements [41,42].

#### Attitude and Self-Efficacy Assessment

Of the 19 studies, 9 measured attitudes or self-efficacy outcomes. Among these, 8 [39,40,43-45,48,49,51] studies demonstrated improvements following AI-based interventions. As with knowledge outcomes, these findings were largely driven by single-group pre-post designs and self-reported measures. Self-efficacy gains were reported using the Communication Confidence Self-Assessment Form (CCSF) [39,45], Self-Efficacy Scale [43], and Patient Clinical Information Exchange and Interprofessional Communication Self-Efficacy Scale (PIE-SES) [48], and communication self-efficacy scores in a mixed methods study [49]. Empathy improvements were measured using the Jefferson Scale of Empathy-Healthcare Providers (JSE-HP) [39]. Attitudes toward opioid overdose were assessed using the Opioid Overdose Attitudes Scale (OOAS) [44]. Student satisfaction and learning confidence were measured using the Student Satisfaction and Self-Confidence in Learning Scale [37], while anxiety was assessed using the Spielberger State-Trait Anxiety Inventory (STAI) [37]. Acceptance of AI-VR interventions was evaluated using a self-designed questionnaire, with scores rising from 3.24 to 4.29 postintervention [40]. Willingness to recommend AI simulations was high (90%‐93%) across diverse learner groups [51]. One study [37] reported that AI simulation induced higher state anxiety compared to standard patient simulation.

#### Technology Acceptance and Usability Assessment

Technology acceptance and usability were evaluated in 6 studies using standardized instruments. The SUS was used in 3 studies [40,44,45], with scores ranging from moderate (61.6 [44], 66.45 [40]) to high (78.56 postintervention [45]). The TAM questionnaire was used in 2 studies [48,49], with perceived usefulness (5.78/7) [48] and immersion (6.06/7) [49] rated highly, while presence scored lower (5.18/7) [49]. The Agent Persona Instrument assessed perceptions of AI doctors, with promoting learning (4.02/5) rated highest and human-likeness (3.12/5) rated lowest [48]. The Chatbot Usability Scale revealed high functionality accessibility (4.44/5) but lower response time satisfaction (3.18/5) [49]. Cognitive load was measured using the NASA-TLX in 2 studies [44,50], with moderate workload reported (47.9/100 [44]; mental demand 10.8/21; and effort 12.7/21 [50]).

#### Implementation Outcomes Assessment

Three studies assessed implementation outcomes using validated measures. The Acceptability of Intervention Measure (AIM), Intervention Appropriateness Measure (IAM), and Feasibility of Intervention Measure (FIM) were used in 2 studies [42,44]. In Liaw et al [42], scores on a 7-point scale ranged from 4.63 (acceptability) to 4.98 (appropriateness). In Swan et al [44], all 3 measures scored above 3.9/5, indicating positive perceptions. One study [42] measured adoption (4.72/7) and overall satisfaction (68.6%). Development costs and time were reported in one study [47], with case development costs ranging from US $5000 to US $9000 and at least 8 hours of faculty time required.

#### Clinical Competency and Performance Assessment

Clinical competency was evaluated in 7 studies using various tools. The CCQ showed significant improvement in one study (Group B improved by 47.68 points; *P*=.02) [38]. The GKCSAF demonstrated significant group×time interaction effects (β=8.96‐9.62; *P*<.001) [39]. Clinical competence was also significantly improved using a standardized clinical performance scale in a quasiexperimental study (Experimental Group 203.76 vs Control Group 171.11; *P*=.02) [41]. Clinical performance was assessed using the Nursing Performance Profile 5 (NPP5), with 60% of participants scoring above 70% [50]. Oral case presentation ability was measured using the Summarize, Narrow, Analyze, Probe, Plan, and Select (SNAPPS) format, with dimension scores ranging from 79% (physical examination) to 93% (subjective summary and differential diagnosis) [46]. Maternal-newborn clinical communication performance was evaluated using the MNCCAF, showing significant improvement from baseline (T0=8.07) to postintervention (T2=9.28; *P*<.001) [45]. The AI Chatbot Assessment Rubric evaluated GPT-patient interactions, with readability scoring highest (2.96/3) and accuracy lowest (2.46/3) [49]. Situational awareness was assessed using the Situation Awareness Global Assessment Technique (SAGAT), with correct response rates ranging from 20% to 100% across questions [50].

#### Qualitative Findings

Qualitative data were collected in 11 studies through focus group interviews [40,45,47-50,52-55], think-aloud protocols [50], debriefing questions [46,47,54], and open-ended feedback [46,49]. Thematic analysis revealed several consistent findings across studies. Pedagogical benefits included enhanced confidence and reduced anxiety [45,52,53,55], improved communication skills and clinical reasoning [47,50,52,54,55], and provision of safe, repeatable, low-pressure practice environments [45,52-54]. Technical limitations were frequently reported, including unnatural or robotic interactions [48,53-55], response delays [47,49], lack of nonverbal cues and physical examination dimensions [50,52-55], and system instability [40,44,50]. Educational design recommendations included introducing AI simulations early in curricula [52,53,55], integrating AI simulations as complementary rather than replacement tools [48,53,54], and improving debriefing methods to address AI-specific behaviors [47,54].

#### Subgroup Analysis by AI Modality

To explore differential effects, findings were narratively synthesized for each of the 4 prespecified AI modalities. GenAI/LLM interventions (n=7) [38,39,43,45-47,49] consistently improved knowledge, clinical competence, communication skills, self-efficacy, and empathy. Learners valued the safe, repeatable practice with real-time feedback but noted robotic dialogue, absent nonverbal cues, and response delays. AI-driven virtual patients/mannequins (n=5) [37,44,52,54,55] showed mixed results: one RCT found AI inferior to standardized patients for psychomotor BSE, while uncontrolled studies reported significant knowledge gains; affective outcomes diverged, with one RCT documenting higher state anxiety with AI yet qualitative studies highlighting reduced anxiety; learners appreciated physiological realism but cited limited emotional expressiveness and absent physical examination. AI-enhanced VR/MR interventions (n=5) [40,48,50,51,53] demonstrated significant knowledge and performance improvements in uncontrolled studies, with moderate to high satisfaction and immersion but frequent technical instability; cognitive load was moderate, and interactions were described as immersive yet mechanical. AI chatbots/tutors (n=2) [41,42] showed nonsignificant knowledge gains when used as adjuncts, with acceptable usability but slower response times. In summary, GenAI/LLM modalities provide the strongest evidence for cognitive and communication outcomes; AI-driven mannequins show mixed effects with concerns about psychomotor skills; AI-VR/MR offers immersive potential limited by technical challenges; and chatbots serve primarily as adjunctive supports with weak independent effects, underscoring the need to align AI modality with specific learning objectives.

#### Synthesis of Quantitative and Qualitative Evidence

The evidence from the quantitative studies and the qualitative studies was synthesized to draw overall conclusions and provide insight to guide the design of future AI-based nursing education interventions. The synthesis findings are presented in Textbox 2 and relate to the study questions.

Textbox 2. **Quantitative, qualitative, and synthesized evidence.**

**How effective are AI-enhanced simulations in improving nursing students’ knowledge, skills, and attitudes?**

**Quantitative evidence:**

Evidence from intervention studies demonstrated that AI-enhanced simulations significantly improved learning outcomes in the majority of studies. Eight studies reported significant knowledge gains; 8 studies showed enhanced self-efficacy or communication confidence; and 4 studies reported moderate to high technology acceptance and usability scores. Interventions may be more effective when they are closely aligned with curriculum objectives, they provide real-time, personalized feedback, students have opportunities for repeated practice, and simulation scenarios demonstrate high clinical authenticity. However, one study found AI-assisted simulation was inferior to standardized patient simulation for breast self-examination skills, and 2 studies reported nonsignificant improvements in critical thinking and knowledge acquisition.

**Qualitative evidence:**

Evidence from qualitative studies and mixed methods studies suggested that AI-enhanced simulations offer multiple pedagogical benefits. Students consistently reported that AI simulations provided safe, nonjudgmental practice environments that enhanced clinical confidence, communication skills, and critical thinking. AI-driven virtual patients enabled structured, repeatable interactions that facilitated the translation of theory into practice. However, qualitative evidence also revealed significant limitations, including interactions perceived as robotic and lacking emotional expression; technical issues such as inaccurate speech recognition and response delays; absence of physical examination dimensions; and system instability. Students recommended introducing AI simulations early in curricula and positioning them as complementary rather than replacement tools.

**Synthesized findings:**

Overall, the evidence suggests that AI-enhanced simulations represent promising innovative tools for nursing education, but their effectiveness is moderated by multiple factors. To optimize learning outcomes, future interventions should consider the following strategies:

(1) Authenticity enhancement: improve the human-likeness of AI interactions by advancing speech recognition technologies, incorporating emotional expression, and integrating nonverbal cues (eg, facial expressions and body movements) to narrow the authenticity gap between AI and standardized patient simulations

(2) Layered instructional design: differentiate AI simulation experiences according to students’ learning stages, menu-based interactions for structured guidance in early stages, and voice-controlled interactions for fostering critical thinking and clinical reasoning in advanced stages

(3) Technical stability optimization: continuously improve response speed, dialog accuracy, and platform stability to minimize technical disruptions that impair learning experiences and ensure simulation fluidity

(4) Curricular integration: position AI simulations as complements to traditional teaching methods (eg, standardized patient simulations, high-fidelity mannequins) rather than substitutes, creating a “stepped” simulation continuum where AI serves for prelearning and basic skills practice, while traditional simulations address advanced interpersonal and affective competencies

(5) Targeted feedback mechanisms: develop AI systems capable of providing immediate, personalized, and structured feedback that encompasses not only quantitative performance scores but also qualitative analyses of clinical reasoning processes and communication strategies, thereby promoting deep reflection

**What are learners’ perceptions and acceptance of AI-enhanced simulations in nursing education?**

**Quantitative evidence:**

Evidence from 8 studies indicated generally high levels of learner acceptance of AI-enhanced simulations. System Usability Scale scores ranged from 61.6 to 78.56, indicating moderate to good usability. Technology Acceptance Model dimensions revealed that students acknowledged the usefulness and intention to use AI simulations, though presence and immersion scores varied across studies. Implementation outcome measures all exceeded 4.6/7, indicating positive perceptions of acceptability, appropriateness, and feasibility. Regarding recommendation intention, 72%‐93% of students expressed willingness to recommend AI simulations to others and a desire to experience more such learning opportunities.

**Qualitative evidence:**

Evidence from qualitative studies and mixed methods studies revealed nuanced learner perspectives on AI simulations. Positively, students valued the safe, low-pressure practice environments, reported enhanced clinical confidence and communication abilities, and appreciated real-time feedback and structured guidance. Students particularly emphasized that AI simulations helped bridge the theory-practice gap and prepared them psychologically and skill-wise for clinical placements. Negatively, students expressed widespread concerns about technical limitations, including AI interactions feeling “mechanical” and “unnatural”; absence of emotional resonance and nonverbal communication; response delays and speech recognition errors disrupting interaction flow; and lack of physical examination dimensions. Students explicitly stated that AI simulations should not completely replace interactions with real patients or standardized patients but rather serve as supplementary tools to traditional teaching methods.

**Synthesized findings:**

Integrating quantitative and qualitative evidence, learners hold generally positive attitudes toward AI-enhanced simulations, recognizing their value for skills training and confidence building, while maintaining clear awareness of their technical limitations. Future design and implementation of AI simulations should consider the following strategies to enhance learner acceptance:

- Manage expectations through clear positioning: explicitly communicate to students the pedagogical objectives of AI simulations and their positioning within the overall curriculum as tools for foundational skills practice and safe trial-and-error, rather than complete substitutes for the full clinical experience involving human interaction. This helps manage learner expectations and reduces disappointment arising from technical limitations.
- Provide hybrid interaction modalities: offer multiple interaction modes tailored to different learning objectives and student preferences. Menu-based interactions suit early learning and structured knowledge acquisition, while voice-controlled interactions benefit advanced communication skills and clinical reasoning training. Allowing students to choose interaction modes based on their needs enhances learning autonomy and satisfaction.
- Blend automated and facilitator-led feedback: combine AI-generated automated feedback with facilitator-guided reflective debriefing. AI provides immediate, objective performance data, whereas facilitators guide students in analyzing these data, exploring the clinical reasoning processes underlying decisions, and discussing strategies for managing anomalous situations encountered during AI simulations.
- Implement phased curriculum integration: introduce AI simulations early in the curriculum, allowing students to become gradually familiar with the technology and build confidence. As learning progresses, gradually increase simulation complexity and authenticity, ultimately transitioning to standardized patient simulations and clinical placements. Such phased integration facilitates a smooth transition to real clinical environments and maximizes the educational value of AI simulations.
- Optimize the authenticity-accessibility balance: while pursuing technological authenticity, ensure system stability and ease of use to avoid student frustration caused by technical failures. Moderate authenticity (eg, incorporating animated virtual characters) may be more acceptable to students than overly realistic but technically unstable systems.

#### Strength of Evidence by Study Design

To calibrate effectiveness claims to methodological rigor, findings were stratified by design tier. Evidence from the 3 RCTs supports short-term gains in communication confidence and structured knowledge but shows limited or inferior effects for tactile psychomotor skills compared to standardized patients. Controlled quasiexperimental studies (n=4) [41-43,54] consistently report moderate improvements in self-efficacy and usability, though confounding remains possible. The 8 [44-51] uncontrolled pre-post/feasibility studies (including mixed methods without control and cross-sectional) provide preliminary, hypothesis-generating data on acceptance and perceived learning gains; however, the absence of comparators precludes causal inference and inflates susceptibility to maturation and testing effects. Consequently, all statements of “significant improvement” in this review should be interpreted as design-contingent rather than definitive evidence of efficacy.

### Publication Bias and Result Distribution

A notable pattern across the included studies was the predominance of statistically significant positive findings: 8 [38,39,43-46,48,50] out of 11 (73%) studies reporting knowledge or skill outcomes and 8 [39,40,43-45,48,49,51] out of 9 (89%) studies reporting self-efficacy or attitude outcomes demonstrated significant improvements. Due to substantial clinical and methodological heterogeneity, formal statistical assessments of publication bias were not feasible. The near-uniform positive pattern, combined with the predominance of small-sample, uncontrolled feasibility studies, suggests a high likelihood of unpublished null or negative results. This potential publication bias weakens the interpretability of all validity conclusions in this review.

## Discussion

### Principal Findings

This mixed methods systematic review synthesizes empirical evidence from 19 studies (n=1253) on AI-driven simulation in nursing education. The principal finding is that AI-enhanced simulations show short-term promise for improving foundational knowledge, clinical reasoning, and communication confidence, particularly when delivering real-time feedback and enabling repeated practice. However, the evidence base is heavily weighted toward uncontrolled or quasiexperimental designs, with only 3 RCTs available. Qualitative data consistently highlight learner appreciation for psychologically safe, repeatable practice environments, alongside persistent concerns regarding technical instability, unnatural interactions, and a learner-perceived “authenticity gap.”

### Comparison With Existing Research

Quantitative findings indicate that AI simulations exert a positive impact on cognitive and affective learning outcomes, particularly in the domains of communication confidence and knowledge acquisition. This aligns with the broader perspective in medical education literature, which posits that the primary advantage of AI lies in its capacity to provide sustained, on-demand deliberate practice, overcoming the limitations associated with traditional high-fidelity manikins or standardized patients. Notably, AI simulations that offer real-time feedback, adaptive scenario progression, and opportunities for repeated practice are associated with stronger learning outcomes, consistent with the principles of deliberate practice and experiential learning [56]. Furthermore, AI simulations foster psychological safety among students, allowing them to make mistakes without fear of judgment from peers or instructors. This factor emerges as a dominant explanation for the significant reductions in anxiety and enhancements in self-efficacy reported across multiple studies. These findings are congruent with prior research indicating that students are more likely to engage boldly in nursing practice training within environments characterized by low error costs and greater inclusivity [57-59].

However, the evidence is not entirely consistent and positive. One study found that AI-assisted simulation was inferior to standardized patient simulation in terms of BSE skills, and 2 other studies reported no significant improvement in critical thinking or knowledge. These discrepancies may stem from differences in simulation fidelity, the sensitivity of outcome measures, or the degree of curriculum integration. Importantly, AI simulation may demonstrate differential effectiveness across various types of learning objectives. Existing evidence suggests that AI simulation shows significant advantages in achieving highly structured learning objectives, such as communication skills and procedural knowledge, while facing notable limitations in objectives requiring high levels of emotional empathy, cultural sensitivity, and complex interpersonal interaction. Kotlyar and Krasman [60] indicate that learners’ perceptions of the feedback source directly influence its acceptance and learning outcomes. In learning tasks intensive in emotion and interpersonal interaction, learners tend to trust human feedback sources perceived as possessing “benevolence” and “integrity” over AI systems perceived as merely “competent.” Furthermore, a network meta-analysis in nursing education revealed that while AI simulation was highly effective in enhancing knowledge acquisition (standardized mean difference=1.11), its effect on cultivating practical skills was relatively limited [61]. Research in the field of social-emotional learning similarly found that AI underperformed compared to human educators in achieving deeper instructional goals, such as facilitating guided reflection and the internalization of social-emotional knowledge [62]. Therefore, educators should select appropriate teaching strategies based on the learning objectives. For highly structured tasks such as foundational communication skills training, history-taking, and health education, AI simulation can provide standardized, repeatable practice opportunities. Conversely, for scenarios requiring emotional empathy, cultural sensitivity, and complex clinical judgment, traditional standardized patient simulation and clinical practicums remain irreplaceable. Moreover, it is essential to gradually enhance the naturalness of interaction and emotional expression while ensuring technical stability and usability, avoiding the sacrifice of the learning experience in pursuit of excessive technical complexity [63].

This finding corroborates the views expressed in some qualitative studies, namely that students perceive interactions with AI as lacking emotional depth, nonverbal communication, and the dimension of physical examination. This observation aligns with the results of a recent qualitative study involving nursing graduate students. Research by Jiang et al [64] found that while nursing students held positive attitudes toward GenAI, they commonly raised concerns regarding content accuracy and technical shortcomings. We term this phenomenon the “authenticity gap.” In this review, the “authenticity gap” is explicitly defined as a learner-perceived discrepancy between AI-driven interactions and human clinical encounters, encompassing deficits in emotional resonance, nonverbal cue recognition, and tactile examination dimensions. It emerges primarily from qualitative usability and experiential data rather than direct performance comparisons. Although AI demonstrates proficiency in handling diagnostic logic and conversational flow, it currently falls short in replicating the tactile and empathetic complexities inherent in human care. Furthermore, the study found that technical malfunctions, such as delays in speech recognition, can disrupt the interaction flow and potentially increase the extraneous cognitive load and state anxiety of some learners. This finding is highly consistent with the conclusions of a recent scoping review published by Chan et al [65]. That review identified barriers to the implementation of AI simulations, including system instability, inaccurate speech recognition, and a lack of structured training for instructors. These shared findings further substantiate that the implementation of AI simulation in education is not merely a matter of technological introduction but involves a systemic transformation encompassing pedagogical philosophy, organizational support, and ethical governance.

Numerous previous studies have indicated that learner acceptance is a critical determinant for the successful application of AI technology in the educational field. The quantitative findings of this review demonstrate that across the included studies, usability, acceptability, and perceived usefulness were consistently rated at moderate to high levels, with overall SUS scores falling within the acceptable range. Dimensions of the TAM further corroborated that students recognized the value of AI simulations for learning, particularly in terms of accessibility and structured guidance. Qualitative evidence enriched these findings, untangling the nuanced ways in which learners experience AI simulations. Students valued the safe, low-risk environment where they could attempt tasks repeatedly without fear of judgment. They also appreciated the consistency and repeatability of AI interactions, which helped bridge the gap between theory and practice. However, persistent concerns were noted regarding the unnaturalness of AI dialogues, the absence of nonverbal cues, and technical instability. These issues highlight the necessity for ongoing improvements in natural language processing and affective expression modeling to enhance the authenticity of AI-driven simulations.

### Implications for Practice and Research

Our review findings highlight the potential of AI simulation in nursing education. In the long term, with the advancement of AI technology, its integration into the daily practice of nursing education is inevitable. However, within the current technological landscape, several barriers to successful implementation remain. To facilitate the integration of AI simulation, based on our review results, we offer several recommendations for nursing educators and instructional designers.

#### Phased Integration

AI simulation should not be viewed as a wholesale replacement for traditional clinical training or standardized patients. Instead, educators should adopt a “stepped” simulation continuum. In the early stages of a curriculum, AI is best suited for prelearning, history-taking practice, and foundational clinical reasoning. As students progress, they should transition to high-fidelity human patient simulators and human standardized patients to develop complex psychomotor skills and advanced emotional intelligence.

#### Scaffolded Interaction Modes

To accommodate different learning stages and reduce frustration, AI systems should offer hybrid interaction modes. Novice learners may benefit from menu-based interactions for acquiring structured knowledge, while advanced students can use open-ended, voice-controlled GenAI features to train clinical adaptability and responses under pressure.

#### The Critical Role of Human-Facilitated Debriefing

While AI can provide immediate, objective performance metrics, it currently lacks the capacity for deep, context-aware reflection. Our findings indicate that it is crucial to combine AI-generated automated feedback with nursing instructor-led reflective debriefing. Human educators remain essential to help students unpack their clinical reasoning processes and address any anomalous AI behaviors encountered during the simulation.

### Strengths and Limitations

The primary strength of this review lies in its mixed methods convergent segregated design. By integrating objective performance data with qualitative learner experiences, we were able to assess not only whether AI simulations work but also how and why they succeed or fail in specific contexts. Furthermore, the inclusion of contemporary GenAI technologies (eg, ChatGPT-driven virtual patients) ensures the findings are highly relevant to the current technological landscape.

Nevertheless, several limitations must be acknowledged. First, the included studies exhibited considerable heterogeneity in terms of AI modalities (screen-based vs VR vs chatbots), intervention durations, and measurement instruments, which precluded a quantitative meta-analysis and limited the ability to determine standardized effect sizes. Second, a significant proportion of the studies relied on pre-post designs lacking active control groups, increasing the risk of bias. Third, all included studies assessed outcomes immediately or within a short postintervention window; no study examined skill retention beyond the immediate educational context or transfer to clinical practice. This constitutes a critical evidence gap. Fourth, a small number of studies included nonnursing learners, which may limit the applicability of some findings to prelicensure nursing education. Fifth, the near-uniform positive result pattern raises concern about publication bias, which cannot be formally tested given study heterogeneity but should be considered when interpreting the overall evidence picture. Sixth, several quantitative studies had small samples, limiting statistical power and precision of effect estimates. Finally, most studies were conducted in high-income countries or regions with robust technological infrastructure; no studies originated from Africa, South America, or low-income countries, and the concentration of studies from Greater China may reflect regional research networks rather than globally representative sampling.

### Conclusion

This systematic review indicates that AI-driven simulations show promise for improving nursing students’ cognitive knowledge, clinical reasoning skills, and communication confidence, particularly by providing a safe, repeatable practice environment. Evidence from RCTs and controlled quasiexperimental studies supports these benefits, though the predominance of uncontrolled designs and the absence of longitudinal data on skill retention and clinical transfer warrant caution in interpreting strength of effectiveness claims. Current evidence suggests AI is most effective for highly structured learning objectives (foundational communication and history-taking) and less effective for advanced psychomotor skills and emotionally complex interpersonal interactions.

Although these positive outcomes demonstrate potential for modernizing nursing instruction, the reported negative technical experiences—captured in the “authenticity gap”—necessitate greater consideration and incorporation of input from both students and educators during the design, implementation, and evaluation phases of AI technologies. The evidence supports positioning AI as a complementary tool within a stepped simulation continuum, alongside traditional methods, rather than as a replacement for standardized patients or clinical placements.

The findings of this review should serve as a starting point for more precise research into AI-enhanced simulation, using standardized measures of clinical competence to obtain a more comprehensive overview. This can be achieved through longitudinal and multicenter RCTs, implementation strategies addressing technical barrier management, further research on integrating automated AI feedback with human-facilitated debriefing, and broader resource evaluation (eg, via cost-effectiveness studies of AI scenario development). By addressing these gaps, the potential of AI-driven simulation in nursing education can be fully realized, improving students’ clinical preparedness and ultimately enhancing patient care outcomes.

## Supplementary material

10.2196/95167 Multimedia Appendix 1 Search strategy.

10.2196/95167 Multimedia Appendix 2 Risk of bias and methodological quality assessment results for all included studies.

10.2196/95167 Multimedia Appendix 3 Summary of key findings from the 19 included studies.

10.2196/95167 Checklist 1 PRISMA checklist.

## Abbreviations

AHRQ: Agency for Healthcare Research and Quality
AIM: Acceptability of Intervention Measure
BSE: breast self-examination
CCQ: Clinical Competence Questionnaire
CCSF: Communication Confidence Self-Assessment Form
FIM: Feasibility of Intervention Measure
GenAI: generative AI
GKCSAF: Gap-Kalamazoo Communication Skills Assessment Form
IAM: Intervention Appropriateness Measure
JBI: Joanna Briggs Institute
JSE-HP: Jefferson Scale of Empathy - Healthcare Providers
LLM: large language model
MMAT: Mixed Methods Appraisal Tool
MNCCAF: Maternal and Newborn Care Communication Assessment Form
MR: mixed reality
NASA-TLX: National Aeronautics and Space Administration Task Load Index
NPP5: Nursing Performance Profile 5
OOAS: Opioid Overdose Attitudes Scale
OOKS: Opioid Overdose Knowledge Scale
PIE-SES: Patient Clinical Information Exchange and Interprofessional Communication Self-Efficacy Scale
PRISMA: Preferred Reporting Items for Systematic Reviews and Meta-Analyses
PROSPERO: International Prospective Register of Systematic Reviews
RCT: randomized controlled trial
RoB 2: risk of bias tool 2
ROBINS-I: Risk of Bias in Nonrandomized Studies of Interventions
SAGAT: Situation Awareness Global Assessment Technique
SNAPPS: Summarize, Narrow, Analyze, Probe, Plan, and Select
STAI: State-Trait Anxiety Inventory
SUS: System Usability Scale
TAM: Technology Acceptance Model
TiDier: Template for Intervention Description and Replication
VR: virtual reality
