# Supplementary material for: AI-Powered Simulation for Nursing Education: Mixed Methods Systematic Review
Source: J Med Internet Res. 2026 Jul 21;28:e95167. doi: 10.2196/95167 (PMC13387420; doi:10.2196/95167)
Supplement: Multimedia Appendix 1 [file jmir-v28-e95167-s001.docx]

Supplementary material 1

Search strategy

# C1: Nursing Education

(“Education, Nursing”[Mesh] OR “Students, Nursing”[Mesh] OR “nursing education” OR “nursing student” OR “nurse education” OR “nursing curriculum” OR “clinical competence” OR “nurse student*” OR “clinical education” OR “nursing school*”)

AND

# C2: Simulation

(“Simulation Training”[Mesh] OR “Computer Simulation”[Mesh] OR “Virtual Reality”[Mesh] OR “Augmented Reality”[Mesh] OR simulat* OR simulation OR “simulation training” OR “simulation-based” OR “high-fidelity simulation” OR “virtual simulation” OR “standardized patient” OR “simulated patient” OR “virtual reality” OR “augmented reality” OR “mixed reality” OR “serious game*” OR “game-based learning” OR “VR” OR “AR”)

AND

# C3: Artificial Intelligence

(“Artificial Intelligence”[Mesh] OR “Machine Learning”[Mesh] OR “Deep Learning”[Mesh] OR “Natural Language Processing”[Mesh] “artificial intelligence” OR AI OR “machine learning” OR “deep learning” OR “natural language processing” OR NLP OR “computer vision” OR “intelligent tutoring system*” OR chatbot* OR “generative AI” OR “large language model*” OR LLM OR ChatGPT OR “intelligent system” OR “intelligent agent” OR “intelligent tutor” OR “conversational agent” OR “virtual patient” OR “adaptive learning”)
