# Supplementary material for: AI-Powered Simulation for Nursing Education: Mixed Methods Systematic Review
Source: J Med Internet Res. 2026 Jul 21;28:e95167. doi: 10.2196/95167 (PMC13387420; doi:10.2196/95167)
Supplement: Multimedia Appendix 2 [file jmir-v28-e95167-s002.docx]

**Supplementary material 2: Results of literature quality evaluation**

Results of Risk Bias Assessment in Randomized Controlled Trials

| Study | 1.Bias arising from the randomization process | 2.Bias due to deviations from intended interventions | 3.Bias due to missing outcome data | 4.Bias in measurement of the outcome | 5.Bias in selection of the reported result | Overall |
| --- | --- | --- | --- | --- | --- | --- |
| Fung et al., (2025) [38] | Low | Some | Low | Low | Low | Low |
| Simsek-Cetinkaya and Cakir (2023) [37] | Some | Low | Low | Some | Low | Some |
| Chen (2025) [39] | Low | Low | Low | Some | Low | Low |

Results of Quality Assessment of Quasi-Experimental Study

| Study | Bias due to confounding | Bias in selection of participants | Bias in classification of interventions | Bias due to deviations from intended interventions | Bias due to missing outcome data | Bias in measurement of outcomes | Bias in selection of the reported result | Overall |
| --- | --- | --- | --- | --- | --- | --- | --- | --- |
| Chang and Su, (2025) [43] | Moderate | Moderate | Low | Low | Low | Low | Low | Moderate |
| Liaw et al., (2025) [42] | Moderate | Moderate | Low | Low | Low | Low | Low | Moderate |
| Park and Kim, (2025) [41] | Low | Low | Low | Low | Low | Low | Low | Low |
| Xiong et al., (2025) [40] | Moderate | Moderate | Low | Low | Low | Low | Low | Moderate |
| Anthamatten et al., (2025) [46] | Moderate | Low | Low | Low | Low | Low | Low | Moderate |
| Chen and Liou, (2025) [45] | Low | Low | Low | Low | Low | Low | Low | Low |
| Swan et al., (2025) [44] | Moderate | Moderate | Low | Low | Low | Low | Low | Moderate |

Results of Quality Assessment of Mixed Methods Study

| **Included studies** | **Screening Questions** | | **Qualitative research score item** | | | | | **Quantitative research score item** | | | | | **Mixed methods research score item** | | | | | **Overall** |
| --- | --- | --- | --- | --- | --- | --- | --- | --- | --- | --- | --- | --- | --- | --- | --- | --- | --- | --- |
|  | **(1)** | **(2)** | **(3)** | **(4)** | **(5)** | **(6)** | **(7)** | **(8)** | **(9)** | **(10)** | **(11)** | **(12)** | **(13)** | **(14)** | **(15)** | **(16)** | **(17)** |  |
| Sepanloo et al., (2025) [50] | Yes | Yes | Yes | Yes | Yes | Yes | Unclear | Yes | Unclear | Yes | Yes | Yes | Yes | Yes | Yes | Yes | Yes | High |
| Kim et al., (2025) [49] | Yes | Yes | Yes | Yes | Yes | Yes | Unclear | Yes | Yes | Yes | Yes | Yes | Yes | Yes | Yes | Yes | Yes | High |
| Liaw et al., (2023) [48] | Yes | Yes | Yes | Yes | Yes | Yes | Unclear | Yes | Yes | Yes | Yes | Yes | Yes | Yes | Yes | Yes | Yes | High |
| McGrew et al., (2025) [47] | Yes | Yes | Yes | No | Yes | Yes | Yes | Yes | No | No | Unclear | Yes | Yes | No | Yes | Yes | No | Moderate |

Note: Evaluation items for “screening questions”: (1) Is the research question clear? (2) Can the collected data answer research questions? Qualitative research evaluation item: (3) Is the method suitable for answering research questions? (4) Is the data collection method sufficient to answer the research question? (5) Is the collected data sufficient to extract research findings? (6) Is their sufficient data to support the explanation of the results? (7) Is their consistency in the source, collection, analysis, and interpretation of data? Quantitative research evaluation item: (8) Is the sampling method appropriate for answering research questions? (9) Is the sample representative of the target population Is the measurement method appropriate? (10) Is the risk of unresponsive offset low? (12) Is the statistical analysis method appropriate? Mixed methods research score item: (13) Is there an adequate rationale for using a mixed methods design to address the research question? (14) Are the different components of the study effectively integrated to answer the research question? (15) Are the outputs of the integration of qualitative and quantitative components adequately interpreted? (16) Are divergences and inconsistencies between quantitative and qualitative results adequately addressed? (17) Do the different components of the study adhere to the quality criteria of each tradition of the methods involved?

Results of quality assessment of cross-sectional study

| Included studies | (1) | (2) | (3) | (4) | (5) | (6) | (7) | (8) | (9) | (10) | (11) | Overall |
| --- | --- | --- | --- | --- | --- | --- | --- | --- | --- | --- | --- | --- |
| De Mattei et al., (2024) [51] | Yes | Yes | Yes | No | No | No | Unclear | Yes | No | Yes | Not applicable | Moderate |

Note: (1) Has the source of the data been clearly identified (survey, literature review)? (2) Have the inclusion and exclusion criteria for the exposed and non-exposed groups (case and control) been listed or referenced from previous publications? (3) Has the time period for identifying patients been provided? (4) If it is not from the population source, are the research subjects continuous? (5) Does the subjective factor of the evaluator conceal other aspects of the research object? (6) Describe any evaluation conducted to ensure quality (such as testing/retesting subjective outcome indicators)? (7) Explained the reasons for excluding any patients from analysis? (8) Described measures for evaluating and/or controlling confounding factors? (9) If possible, explain how lost data is handled in the study? (10) Summarized the response rate of patients and the completeness of data collection? (11) If there is follow-up, the percentage of incomplete patient data or follow-up results should be specified?

Results of Qualitative Study Quality Evaluation

| Included studies | (1) | (2) | (3) | (4) | (5) | (6) | (7) | (8) | (9) | (10) | Overall |
| --- | --- | --- | --- | --- | --- | --- | --- | --- | --- | --- | --- |
| Harder et al., (2023) [54] | Yes | Yes | Yes | Yes | Yes | No | No | Yes | Yes | Yes | Moderate |
| Carlos Martinez et al., (2025) [55] | Yes | Yes | Yes | Yes | Yes | Yes | Yes | Yes | Yes | Yes | High |
| Shorey et al., (2020) [53] | Yes | Yes | Yes | Yes | Yes | No | No | Yes | Yes | Yes | Moderate |
| Teixeira et al., (2024) [52] | Yes | Yes | Yes | Yes | Yes | Yes | Yes | Yes | Yes | Yes | High |

Note: (1) Is the philosophical foundation consistent with methodology? (2) Is methodology consistent with research questions or objectives? (3) Is the methodology consistent with the data collection method? (4) Is the representativeness and typicality of methodology and data consistent with the methods of data analysis? (5) Is the methodology consistent with the interpretation of results? (6) Does it explain the researcher’s own situation from the perspective of cultural background and values? (7) Has the impact of the researcher on the research been explained, or has the research had an impact on the researcher? (8) Is the research object and its viewpoint typical? (9) Has the research been approved by the ethics committee? (10) Does the conclusion come from the analysis and interpretation of the data.
