# Supplementary material for: AI-Powered Simulation for Nursing Education: Mixed Methods Systematic Review
Source: J Med Internet Res. 2026 Jul 21;28:e95167. doi: 10.2196/95167 (PMC13387420; doi:10.2196/95167)
Supplement: Multimedia Appendix 3 [file jmir-v28-e95167-s003.docx]

Supplementary material 3: Key findings of the studies included in the review

|  | Study  (Author, Year) | Primary outcome measures | Key Findings (Qualitative) | Key Findings (Quantitative) | Author’s conclusion |
| --- | --- | --- | --- | --- | --- |
| 1 | Simsek-Cetinkaya and Cakir (2023) [37] | BSE Skill Score, Student Satisfaction, Learning Confidence, State Anxiety | / | BSE Skill Score: AI group 59.71 ± 12.01, SPS group 73.72 ± 15.53 (p<0.05);  Satisfaction Score: AI group 56.59 ± 6.27, SPS group 50.45 ± 12.19 (p<0.05);  State Anxiety Score: AI group 40.82 ± 8.17, SPS group 36.50 ± 8.23 (p<0.05) | AI simulation can improve student satisfaction, but it is not as effective as standard patient simulation in enhancing BSE skills and may induce higher levels of anxiety. It is recommended to use it as a supplementary teaching method. |
| 2 | Fung et al., (2025) [38] | Clinical Competence: Clinical Competence Questionnaire (CCQ);  Cultural Awareness: Cultural Awareness Scale (CAS);  AI Readiness: Medical Artificial Intelligence Readiness Scale for Medical Students (MAIRS-MS);  Simulation Effectiveness: Simulation Effectiveness Tool – Modified (STE-M) (measured only at T1) | / | Total CCQ score: Group B T1 increased by 47.68 points, Group A T1 increased by 24.95 points, with an inter-group difference of 16.59 points (p=0.020);  MAIRS-MS: Group B improved by 30.18 points, Group A improved by 16.64 points, with an inter-group difference of 12.09 points (p=0.003);  SET-M: 75% of students considered debriefing helpful for learning; 68.2% felt more confident in their nursing assessment skills | GenAI simulation shows potential in enhancing clinical competence and AI readiness, particularly as an initial teaching tool; both types of simulation are effective, and a combined approach is recommended to optimize learning outcomes |
| 3 | Chen, (2025) [39] | Empathy: JSE-HP Scale (20 items, 7-point Likert);  Communication Confidence: CCSF Scale (11 items, 5-point Likert);  Communication Skills: GKCSAF Scale (9 items, 5-point Likert) | / | JSE-HP Empathy Score (T3): Experimental group 110.57 vs. control group 103.71. GEE analysis showed a significant group × time interaction effect (T1: β=16.88, p<.001; T2: β=10.53, p<.001; T3: β=9.08, p<.001).  CCSF Communication Confidence (T3): Experimental group 48.08 vs. control group 39.98. The group × time interaction effect was significant (T1: β=8.56, p<.001; T2: β=6.89, p<.001; T3: β=7.36, p<.001).  GKCSAF Communication Skills (T3): Experimental group 39.55 vs. control group 29.71. The group × time interaction effect was significant (T1: β=9.62, p<.001; T2: β=8.38, p<.001; T3: β=8.96, p<.001). | As an innovative teaching tool integrating generative AI and VR, NLP-VRCS can effectively improve nursing students' empathy, communication confidence and communication skills, and is particularly suitable for obstetric nursing education. |
| 4 | Xiong et al., (2025) [40] | Acceptability (Self-designed Questionnaire), System Usability Scale (SUS), Qualitative Interview Topics | Participants gave positive evaluations of the immersion and interactivity of VR, but expressed concerns about the complexity of device operation and system stability; the potential of the integration of AI and VR was recognized. | Acceptance score: 3.24 before intervention → 4.29 after intervention (p<0.001)  SUS usability score: 66.45 (moderate usability)  Paired t-test showed significant improvement in all dimensions (p<0.001) | AI+VR escape room teaching method can significantly improve nurses' and midwives' acceptance of disaster emergency training, demonstrating favorable educational potential and promotion value. However, further optimization of the interface and system stability is required. |
| 5 | Park and Kim, (2025) [41] | Obstetric nursing knowledge, critical thinking tendency, clinical competence, digital literacy | / | Obstetric Nursing Knowledge: Experimental Group 14.78 vs. Control Group 12.20 (t=7.03, p<.001)  Clinical Competence: Experimental Group 203.76 vs. Control Group 171.11 (t=7.80, p=.020)  Digital Literacy: Experimental Group 82.00 vs. Control Group 71.23 (t=4.18, p<.001)  Critical Thinking: Experimental Group 62.31 vs. Control Group 58.73 (t=1.77, p=.098, not significant) | Scenario-based simulation teaching assisted by AI tutors can significantly improve obstetric knowledge, clinical competence and digital literacy among nursing students, whereas the enhancement of critical thinking requires longer-term interventions; it is recommended to promote AI-assisted simulation in obstetrics and gynecology nursing education. |
| 6 | Liaw et al., (2025) [42] | Knowledge of clinical deterioration identification and response, interprofessional communication knowledge, implementation outcomes (acceptability, appropriateness, feasibility, adoption) | / | Knowledge Score (Recognition and Response): Experimental group 18.0 vs. Control group 17.9 (p=0.78, not significant)  Communication Knowledge: Experimental group 4.1 vs. Control group 3.9 (p=0.26, not significant)  Implementation Outcomes (7-point scale): Appropriateness 4.98, Feasibility 4.65, Adoption 4.72, Acceptability 4.63  68.6% of students were satisfied with the overall experience, and 73.8% considered AI-enabled VRS useful | As a supplement to face-to-face simulations, AI-enabled VRS does not significantly improve knowledge scores, yet students have given positive evaluations of its educational value. In the future, generative AI (such as ChatGPT) should be integrated to enhance the realism of interaction, and technical stability should be optimized to facilitate sustainable implementation. |
| 7 | Chang and Su, (2025) [43] | Obstetric care knowledge, self-efficacy, problem-solving ability, decision-making ability | / | Knowledge score: experimental group 85.45 vs. control group 77.58 (t=2.61, p<.05)  Self-efficacy: experimental group 4.74 vs. control group 3.85 (t=6.28, p<.001)  Problem-solving ability: experimental group 4.58 vs. control group 3.92 (t=4.05, p<.001)  Decision-making ability: experimental group 4.46 vs. control group 3.82 (t=4.14, p<.001) | GAI-PCC teaching strategy can significantly improve nursing students' obstetric knowledge, self-efficacy, problem-solving skills, and decision-making abilities. It is an effective and innovative teaching method that is worthy of promotion in nursing education. |
| 8 | Swan et al., (2025) [44] | Feasibility (SUS, NASA TLX), acceptability (AIM, IAM, FIM), opioid overdose knowledge (OOKS), attitude (OOAS) | / | SUS usability score: 61.6 (below the 68 average line)  AIM: 3.9/5, IAM: 4.1/5, FIM: 4.1/5  NASA TLX load: 47.9/100  OOKS knowledge: baseline 18.2 → 20.3 after one month (p=0.004)  OOAS attitude: baseline 113.4 → 117.7 after one month (p=0.002) | Incorporating AI simulated humans into simulation teaching is feasible and acceptable, which can improve students' knowledge and attitudes towards opioid overdose. However, the technical stability needs to be optimized. In the future, it can be expanded to be used in sensitive topics and complex health education scenarios. |
| 9 | Chen and Liou, (2025) [45] | Communication confidence (VAS-CSC), maternal and infant care communication skills (MNCCAF), system usability (SUS), focus group interviews | The system provides real-time feedback, highly realistic scenarios, and a stress-free learning environment, helping students transform theory into practice and reducing anxiety during clinical internships. | Communication confidence: T0 5.56 → T1 7.14 (d=1.44) → T2 7.64 (d=1.92), GEE analysis showed a significant improvement (p<.001)  Communication skills: T0 8.07 → T1 9.17 (d=0.93) → T2 9.28 (d=1.14), GEE significant (p<.001)  SUS usability: T0 34.66 → T1 78.56 (d=6.08) → T2 76.58 (d=6.38), GEE significant (p<.001) | ChatVR-ONCS integrates AI and VR technologies, significantly enhancing nursing students' communication confidence and skills, with the effects lasting for 3 months. The system has high usability and receives positive feedback from students, making it an effective and innovative tool for obstetric nursing education. |
| 10 | Anthamatten et al., (2025) [46] | Oral case presentation skills (scores of each dimension of SNApps), post-case test scores, students’ perceived feedback | Students generally believe that AI-driven simulations have improved their communication skills, critical thinking, and clinical reasoning abilities; students self-assessed that their oral presentation skills have improved by 20%. | Overall average score: 89% (n=82)  SNApps scores by dimension: Subjective summary 93%, Physical examination 79% (adjusted due to system limitations), Clinical reasoning 85%, Differential diagnosis 93%, Diagnostic analysis 93%, Questions to mentors 84%, Initial plan 81%, Self-directed learning 85%  Average score of post-case quizzes: 86.6%  Scores of standardized patient presentations at the end of the semester all improved | AI-driven virtual simulations provide students with safe and repeatable opportunities to practice oral case presentations. Combined with structured feedback, they effectively enhance students' clinical reasoning and communication skills, making them an innovative tool for competency-based assessment. |
| 11 | Sepanloo et al., (2025) [50] | Situation awareness (SAGAT score), cognitive load (NASA-TLX), clinical ability (NPP5 performance), user feedback (MR experience survey), qualitative analysis | Participants can identify key patient needs (such as fluid resuscitation, oxygenation, and infection control) and demonstrate good clinical reasoning skills. However, there are technical limitations (such as issues with heart sound activation) and a need for more structured guidance. Common errors include failure to verify patient identity, failure to wash hands/put on gloves, and setting the oxygen flow rate too low. | SAGAT accuracy rate: Question 1 30%, Question 2 80%, Question 3 100%, Question 4 20%, Question 5 60%  NPP5 performance: 50-60% (1 person), 60-70% (2 people), 70-80% (3 people), 80-90% (3 people), 90-100% (1 person); those with rich experience scored higher  NASA-TLX (21-point scale): Mental demand 10.8, Physical demand 5.7, Temporal demand 10.7, Effort 12.7, Performance 12.8, Frustration 10.1  The average score of the MR experience survey is 4-5 points (5-point scale), indicating that the system is useful, easy to use and realistic | AI-enhanced mixed reality training platform can effectively support nursing learners in learning in complex clinical scenarios, improving their situational awareness and clinical reasoning abilities. The system is flexible and accessible, and more realistic than traditional simulations. In the future, larger sample studies are needed, and sensor technology should be integrated. |
| 12 | Kim et al., (2025) [49] | Usability (Chatbot Usability Scale), Perceived Virtual Learning Environment (TAM-based 7-dimensional scale), Communication Self-Efficacy, Open-ended Questions, GPT-Patient Dialogue Evaluation (Accuracy, Safety, Relevance, Readability) | Educational effects include: a sense of reality and immersion, objective data supporting learning, effective communication practice, and interest in new learning methods. Technical limitations include: response delay, limited interaction (lack of movement), unnatural AI voice, and the need for more scenarios. | Communication self-efficacy: 61.57 ± 4.58 → 64.32 ± 5.54 (t=-2.82, p=.009)  Usability (5-point scale): functional accessibility 4.44, functional quality 4.10, dialogue quality 4.15, privacy and security 4.07, response time 3.18  Virtual learning environment (7-point scale): immersion 6.06 (highest), sense of presence 5.18 (lowest)  Dialogue evaluation (3-point scale): readability 2.96 (highest), accuracy 2.46 (lowest) | GPT-Virtual Patient can enhance communication self-efficacy, provide a high sense of immersion and functional accessibility, but it needs to improve response time, interaction limitations, and accuracy, and develop more scenarios; in the future, it is necessary to expand the sample size and adopt more rigorous designs to verify the effect. |
| 13 | Liaw et al., (2023) [48] | Communication knowledge (8-question test), interdisciplinary communication self-efficacy (PIE-SES scale), virtual environment perception (TAM questionnaire), AI doctor perception (Agent Persona Instrument), focus group interviews | Theme 1: Related to the real world - AI scenarios help review clinical knowledge and communication skills;  Theme 2: AI vs. Human Intelligence - AI doctors provide more structured guidance, but their communication is stiff and mechanical;  Theme 3: Complementary to face-to-face learning - it is suggested that AI-VRS be used as a pre-learning resource for face-to-face simulations | Communication knowledge: Pre-test 4.22 → Post-test 5.59 (p < .001)  Self-efficacy: Pre-test 56.9 → Post-test 64.7 (p < .001)  TAM (7-point scale): Perceived usefulness 5.78, Usage intention 5.75, Confirmation 5.71, Ease of use 5.46, Satisfaction 5.01, Presence 4.51, Immersion 3.78  Perception of AI doctor (5-point scale): Facilitation of learning 4.02, Credibility 3.82, Anthropomorphism 3.12 (the lowest), Attractiveness 3.50 | AI-enabled VRS can effectively enhance nursing students' knowledge of interprofessional communication and self-efficacy. Students have a high evaluation of AI doctors in promoting learning, but the anthropomorphism and sense of immersion need to be improved. It is suggested that it be used as a supplementary tool for face-to-face simulation to optimize learning effects. |
| 14 | McGrew et al., (2025) [47] | Feasibility of simulation, cultural authenticity, student experience, development cost and time, effectiveness of debriefing | Students enjoy interacting with AI avatars, and response delays are beneficial for thinking; AI avatars can convey emotions through wording, facial expressions, and body movements; The occurrence of non-programmed responses from AI (such as mentioning unexpected social support) triggers concerns about abuse; Debriefing needs to be improved based on AI's abnormal behaviors; Teachers believe that interactions with AI avatars vary depending on students' questions. | Case development cost: $5,000 - $9,000 per case  Development time: At least 8 hours of teacher time  Student feedback: Positive evaluations on the pre-brief and telemedicine lectures, believing that debriefing needs to be more in line with the AI modality | Generative AI simulation provides midwifery students with efficient, flexible, person-centered, and culturally responsive opportunities for telemedicine practice. It allows exposure to diverse patient populations and overcomes the limitations in recruiting standardized patients. However, continuous improvements in cultural authenticity and debriefing methods are needed. |
| 15 | Carlos Martinez et al., (2025) [55] | Students’ Views and Experiences with AI-Driven VR Simulations (Collected through Focus Groups) | Extract five themes:  1) Confidence building – Simulation enhances students’ confidence in conducting nursing assessments and clinical practices;  2) Communication skills – Helps students improve their questioning methods, use concise language, and enhance listening and observation abilities;  3) Information collection – Assists in the assessment process and guides the order of questioning, but lacks the physical dimension of physical examinations;  4) Innovative tools and technology acceptance – Students consider VR-AI as an innovative teaching tool, but the limitations of speech recognition and the lack of non-verbal cues need improvement;  5) Knowledge enrichment and self-reflection – Automatically generated feedback promotes the expansion of clinical knowledge and reflection, and it is suggested to introduce it into the curriculum early. | / | AI-driven virtual patient simulation is an innovative and engaging supplementary teaching method that helps develop students' confidence, communication skills, nursing assessment abilities, and readiness for clinical practice. However, it is necessary to continuously improve technologies (such as speech recognition and non-verbal cues) and integrate them into the curriculum. |
| 16 | Harder et al., (2025) [54] | Students’ perceptions of a sense of reality, psychological safety, and skill development (communication, confidence) (through focus group interviews) | 1) Sense of reality: SP simulation provides life-like interactions and non-verbal cues, but the performance of actors varies; AI-VR offers immersive and adaptive conversations, but lacks physical interaction and sometimes presents a robotic feel.  2) Psychological safety: AI-VR provides a non-judgmental and low-pressure practice environment; SP simulation sometimes increases pressure due to the presence of peers/teachers.  3) Strengthening practice: In terms of communication, SP simulation promotes emotional connection, while AI-VR offers structured iterative practice; in terms of confidence, both simulations enhance self-confidence through repeated practice. | / | AI-VR and SP simulation each have their own advantages and should be used in an integrated manner: SP simulation cultivates interpersonal communication and emotional engagement, while AI-VR provides a structured, low-risk practice environment, which is conducive to quick decision-making and repeated practice. It is recommended that the two models be integrated into curriculum design and that teacher training be strengthened. |
| 17 | De Mattei et al., (2024) [[51] | Perceptions of the realism of AI-VSP, perceptions of improved diagnostic capabilities, willingness to recommend, and willingness to use it in the future (collected through Likert scales, Yes/No questions, and open-ended questions) | / | Realism perception (agreement rate): FNP 50%, PA 16.1%, BSN 62.7%, ABSN 87%  Completeness of diagnosis and treatment plan (agreement rate): FNP 41%, PA 51.6%, BSN 65%, ABSN 82%  Improvement in diagnostic ability (agreement rate): FNP 72.7%, PA 74%, BSN 72%, ABSN 89.7%  Willingness to recommend (agreement rate): FNP 90.9%, PA 84%, BSN 93%, ABSN 90%  Help with medical history collection and differential diagnosis (Yes rate): FNP 77%, PA 51.6%, BSN 81%, ABSN 87%  Willingness to try more AI-VSP (Yes rate): FNP 91%, PA 83.8%, BSN 93%, ABSN 89.7% | AI-VSP is widely accepted by students, and it has shown potential as a supplementary learning tool, especially during the COVID-19 pandemic, being able to enhance students' confidence and history-taking abilities. However, the technology still needs improvement (such as in language recognition and emotional expression), and it should serve as a supplement rather than a replacement for traditional teaching methods. |
| 18 | Shorey et al., (2020) [53] | Results of qualitative thematic analysis (students’ user experience, clinical teachers’ evaluation of students’ communication skills) | 1) Students’ attitudes: Worthwhile but with a sense of frustration (due to technical limitations);  2) Role of VP: Enhancing preparation and confidence, practicing structured communication skills;  3) Suggestions for improvement: Enhancing realism (improving speech recognition, increasing emotional expression, non-verbal cues), introducing it early in the curriculum, ensuring continuous accessibility;  4) Evaluation by clinical teachers: Students are effective communicators, but this is influenced by personal traits and experience;  5) Improving communication methods: Increasing situational sensitivity, overcoming language barriers;  6) Technical value: Providing an accessible, safe, and non-judgmental practice environment. | / | Students have mixed attitudes towards interactions with Virtual Patients (VP). They recognize the potential of VP simulations, but improvements are needed regarding the lack of authenticity and technical limitations. It is suggested that VP be used as a supplementary tool rather than a substitute, introduced early in the curriculum, and continuously optimized. |
| 19 | Teixeira et al., (2024) [52] | Students’ experience with two AI-VR interaction methods (collected through focus groups) | Four themes are extracted:  1) Technical literacy: Voice control is more intuitive and natural, but it requires repeating and restating questions; menu-based systems provide structured questions but have too many options.  2) VR as a learning tool: Voice control is more realistic and promotes communication and critical thinking; menu-based systems offer structured guidance but may lose the sense of process.  3) Learning path: Voice control facilitates “learning in action” (communication, critical thinking), and both methods provide “learning from action” (automatic feedback promotes reflection).  4) Transition to independent practice: Both methods enhance confidence, listening skills, communication skills, and critical reasoning, which help prepare for independent practice. | / | VR and AI patient technologies provide pre-registration nursing students with an immersive problem-oriented learning environment, which helps develop various skills in a safe setting. Each of the two interaction methods has its own advantages: voice control is more intuitive and natural, suitable for advanced learning; menu-based interaction offers structured guidance, suitable for the early learning stage. It is recommended to integrate both methods according to the students' learning stages. |

BSE = Breast Self-Examination; CCQ = Clinical Competence Questionnaire; CAS = Cultural Awareness Scale; MAIRS-MS = Medical Artificial Intelligence Readiness Scale for Medical Students; SET-M = Simulation Effectiveness Tool – Modified; GenAI = Generative Artificial Intelligence; VR = Virtual Reality; NLP-VRCS = Natural Language Processing Virtual Reality Communication Simulation; JSE-HP = Jefferson Scale of Empathy–Healthcare Providers; CCSF = Communication Confidence Self-assessment Form; GKCSAF = Gap-Kalamazoo Communication Skills Assessment Form; GEE = Generalized Estimating Equations; SUS = System Usability Scale; AI= Artificial Intelligence; GAI-PCC = Generative Artificial Intelligence-based Patient Character Creation; OOKS = Opioid Overdose Knowledge Scale; OOAS = Opioid Overdose Attitudes Scale; AIM/IAM/FIM = Acceptability/Appropriateness/Feasibility of Intervention Measure; ChatVR-ONCS = ChatGPT-based Virtual Reality Obstetric Nursing Communication Simulation System; VAS-CSC = Visual Analogue Scale for Communication Self-Confidence; MNCCAF = Maternal and Newborn Care Communication Assessment Form; TAM= Technology Acceptance Model; SAGAT= Situation Awareness Global Assessment Technique; NASA-TLX= NASA Task Load Index; NPP5= Nursing Performance Profile 5; MR= Mixed Reality; SPS = Standard Patient Simulation; AI-VSP= Artificial Intelligence Virtual Simulated Patient; PIE-SES= Patient Clinical Information Exchange and Interprofessional Communication Self-Efficacy Scale;
